# Tracking Global Transmission Dynamics of the Plasmid-Mediated *mcr* Gene: A Genomic Epidemiological Analysis

**DOI:** 10.3390/microorganisms14010028

**Published:** 2025-12-22

**Authors:** Jinzhao Long, Xin Wang, Mengyue Liu, Jie Wu, Haiyan Yang, Shuaiyin Chen, Guangcai Duan

**Affiliations:** Department of Epidemiology, College of Public Health, Zhengzhou University, No. 100 Kexue Avenue, Zhengzhou 450001, China; ljz069@zzu.edu.cn (J.L.);

**Keywords:** *mcr* gene, plasmid, horizontal gene transfer, clonal spread, genomic epidemiology

## Abstract

The emergence and spread of mobile colistin resistance (*mcr*) genes pose a significant challenge in controlling multidrug-resistant Gram-negative pathogens. Understanding the epidemiology of *mcr*-carrying plasmids is essential for mitigating their dissemination across humans, animals, and the environment. To characterize their spatiotemporal dynamics on a global scale, we analyzed an extensive collection of 5,549 *mcr*-carrying plasmids spanning 1995 to the present. We found that cross-genera transmission patterns of *mcr*-carrying plasmids varied across four distinct periods. Initially, IncHI2/HI2A plasmids provided a survival advantage across genera and regions, followed by IncI2, and ultimately by IncX4. Moreover, the three plasmid lineages (i.e., IncX4, IncI2, and IncHI2/HI2A) have reached a stable distribution across diverse bacterial hosts and geographic regions through horizontal gene transfer and clonal expansion. By integrating sequence similarity clustering of plasmids and *mcr*-related genetic environments, we identified 79 cross-genus, 43 intra-*E. coli*, and 10 intra-*S. enterica* transmission units. Molecular dating analysis traced the origin of IncX4 plasmids to 1990 in animal hosts, with phylogenetic evidence indicating potential cross-host, -genus, and -region exchange. Notably, IncP1 plasmids emerged as important vectors of *mcr-1* and *mcr-3* spread, particularly in Southeast Asia, warranting enhanced surveillance. These findings provide critical insights into the global transmission networks of plasmid-mediated *mcr* genes and underscore the urgent need for coordinated interventions.

## 1. Introduction

Bacterial antimicrobial resistance (AMR) is a critical problem for human health worldwide [1]. The increasing AMR crisis stems largely from the misuse and overuse of antimicrobial agents in clinical and agricultural settings [2,3]. This has led to the emergence and transmission of multidrug-resistant (MDR), extensively drug-resistant (XDR), and even pandrug-resistant (PDR) pathogens [4]. Of particular concern are carbapenem-resistant pathogens, which show a marked increase in attributable mortality [5]. To combat such infections, colistin has been reintroduced as a last-line treatment option, despite its well-documented nephrotoxicity and neurotoxicity [6,7]. Unfortunately, the growing dependence on colistin has accelerated the emergence of colistin-resistant strains, further complicating treatment strategies [8,9]. Moreover, the use of colistin as a growth promoter in agricultural settings has further increased the risk of colistin resistance [10].

Initially, colistin resistance was attributed to mutational and regulatory changes of chromosomal genes, including *mgrB*, *pmrA*/*pmrB*, *phoP*/*phoQ*, and *pmrC* genes [11,12]. However, this paradigm shifted dramatically in 2015 with the discovery of the mobilized colistin gene *mcr-1* in the *Escherichia coli* (*E. coli*) plasmid from China [13]. The *mcr-1* gene encodes a phosphoethanolamine (pEtN) transferase that confers colistin resistance by conveying pEtN from the cytomembrane to its target, lipid A [14]. Unlike chromosomal-mediated resistance, plasmid-borne *mcr* genes can disseminate rapidly across bacterial species and ecological niches via horizontal gene transfer (HGT) [14]. To date, ten distinct *mcr* variants (*mcr-1* to *mcr-10*) have been identified [15]. The primary bacterial hosts for *mcr* variants are *Enterobacterales*, mainly including *E. coli*, *Salmonella enterica* (*S. enterica*), *Enterobacter cloacae* (*E. cloacae*), and *Klebsiella pneumoniae* (*K. pneumoniae*) [14,16]. Epidemiological surveillance has shown that these *mcr* variants are carried by over 20 types of plasmids [14,17]. In addition to horizontally disseminating antibiotic resistance genes (ARGs) across species boundaries through HGT, plasmids can also vertically transmit them to daughter cells during bacterial replication [18]. The clonal transmission driven by high-risk clones carrying plasmid-borne *mcr* genes has been frequently reported, such as *K. pneumoniae* ST15 [19], *E. coli* ST10 [20], *Enterobacter hormaechei* (*E. hormaechei*) ST78 [21], and *S. enterica* ST34 [22]. Given the contribution of plasmids to *mcr* propagation, several investigations have explored epidemiological characterization of *mcr*-positive plasmids across humans, animals, and environments [17,23]. However, these investigations were based on point surveillance data or relatively limited plasmid sequences. The sharing and public availability of large-scale genomic data provide an opportunity to comprehensively understand the spatiotemporal transmission patterns of *mcr*-positive plasmids on a global scale.

Here, we systemically compiled the most extensive global collection of 5,549 *mcr*-carrying plasmids to date by integrating short-read and long-read sequencing assemblies from three databases. Utilizing this comprehensive dataset, we characterized the spatiotemporal dynamics of *mcr*-positive plasmids across six continents from 1995 to present, delineated the role of HGT and clonal spread events in *mcr* dissemination, and reconstructed the transmission history of the predominant plasmid lineage (i.e., the IncX4 plasmid). This study aims to unveil the current landscape of plasmid-mediated *mcr* transmission on a global scale and to provide new insights into the monitoring and control of *mcr* spread.

## 2. Methods and Materials

### 2.1. Genome Collection

As of 30 April 2024, a total of 13,605 *mcr*-positive genome assemblies were retrieved and downloaded from the NCBI Pathogen Detection database (https://www.ncbi.nlm.nih.gov/pathogens/, accessed on 30 April 2024) using the search criteria “AMR_genotypes: *mcr*”. All the genomes were reassessed by QUAST v.5.2.0 [24] and CheckM v1.1.10 [25]. Low-quality genomes were excluded based on the following criteria: number of contigs ≤ 1000, N50 length ≥ 10,000 bp, completeness ≥ 95%, and contamination ≤ 5%. After data trimming, a total of 13,344 *mcr*-positive genomes were included for subsequent analysis. The detailed metadata for each genome were retrieved from the NCBI database using a local Python script (https://github.com/ljzzzu/Extract_metadata, accessed on 30 April 2024). Detailed information was presented in Table S1.

### 2.2. Determination and Curation of Plasmid-Derived Contigs

To construct a dataset of *mcr*-positive plasmids as large as possible, we combined data from the NCBI Pathogen Detection database, NCBI RefSeq database (https://ftp.ncbi.nlm.nih.gov/refseq/release/plasmid/, accessed on 30 April 2024), and PLSDB database (https://ccb-microbe.cs.uni-saarland.de/plsdb, accessed on 30 April 2024). Firstly, 11,703 high-quality genomes were selected from 13,344 *mcr*-positive genomes identified in the NCBI Pathogen Detection database (number of contigs ≤ 200 and N50 length ≥ 50,000 bp). Subsequently, *mcr*-positive contigs were extracted from the 11,703 high-quality genomes by using ResFinder v4.1 (https://github.com/genomicepidemiology/resfinder, accessed on 30 April 2024). Given that most *mcr* genes were identified on fragmented assemblies resulting from short-read sequencing, we employed a machine-learning tool (RFplasmid v0.0.18: https://github.com/aldertzomer/RFPlasmid, accessed on 30 April 2024) to predict whether the *mcr* genes were located on chromosomal or plasmid sequences. Additionally, we downloaded 86,009 and 59,895 plasmids with “complete” assembly level from the NCBI RefSeq and PLSDB plasmid databases, respectively (access date: 30 April 2024). Similarly, ResFinder v4.1 was used with default parameters to identify *mcr*-positive plasmids. In cases where sequences of the same plasmid were redundantly recorded across the three databases, the plasmid sequence with the highest genome sequencing coverage was retained. Furthermore, PlasmidFinder v2.2.1 was utilized to identify plasmid replicons, and Mob-suite v3.19 (https://github.com/phac-nml/mob-suite, accessed on 30 April 2024) was applied to assign plasmid clusters and predict plasmid mobility. Ultimately, a total of 5,549 *mcr*-positive contigs were included for final plasmid analysis based on the following criteria: (i) plasmid localization predicted by RFplasmid; (ii) at least one plasmid replicon predicted by PlasmidFinder; and (iii) a plasmid cluster ID assigned by Mob-suite (Table S2). The whole analysis flowchart was shown in Figure S1.

### 2.3. Genome Annotation, MLST Analysis, and Core Genome SNP Analysis

In silico MLST analysis was complemented by MLST v.2.23.0 for 13,344 *mcr*-positive genomes (https://github.com/tseemann/mlst, accessed on 30 April 2024), using the corresponding typing scheme for each species. GrapeTree v0.1.8 was employed to construct the minimum spanning tree based on the allelic profiles of MLST (https://github.com/achtman-lab/GrapeTree, accessed on 30 April 2024). For the genomes of 346 ST10 *E. coli* and 248 ST34 *S. enterica* genomes, the “gff” files generated by Prokka v.1.1.13 were applied to obtain the core genome SNPs through Roary v.3.13.0 [26]. Pairwise SNP matrices were calculated using Snp-dists v.0.6 (https://github.com/tseemann/snp-dists, accessed on 30 April 2024), and genome clustering based an SNP threshold (*n* = 20) was performed with GraphSNP [27].

### 2.4. Genetic Environment Determination and Transmission Network Construction

To determine the genetic environments of *mcr* genes, 10 kb of sequences upstream and downstream of each *mcr* gene were extracted from the 5,549 *mcr*-positive plasmids. Firstly, these flanking sequences were clustered by CD-HIT with a 99% identity threshold. The representative flanking sequences for each cluster were re-annotated by Prokka v.1.1.13. To further reduce redundancy, the results were manually reviewed based on previously described genetic environments. A potential transmission unit was defined as a combination of an *mcr* variant, a secondary cluster inferred by Mob-suite, and genetic environments. The contigs without collection year and collection country were excluded. The Python package Networkx was used to visualize the transmission network.

### 2.5. Evolutionary Analysis

The 173 IncX4 plasmids with the assembly level of “complete” were used for inferring the evolutionary history of IncX4 plasmids. The alignment of core genes was implemented through Roary v.3.13.0 [26]. The recombination regions of core genome alignments were filtered by Gubbins v2.3.4 (https://github.com/nickjcroucher/gubbins, accessed on 30 April 2024). The evolutionary processing was illustrated using BEAST v1.10.4 [28]. To evaluate the temporal signal in the ML tree and ensure its reliability, a root-to-tip regression analysis was conducted with Tempest 1.5.3. An HKY85 substitution model and an uncorrelated log-normal relaxed clock were selected to calculate the divergence time. Tracer v1.7.2 was used to assess the convergence, ensuring that all relevant parameters reached an effective sample size > 200. Finally, we use TreeAnnotator v1.10.4 to best fit the posterior tree of the model to provide an annotated MCC tree. FigTree v1.4.4 was used to visualize the results. Bayesian phylogeography was carried out by SpreaD3.

## 3. Results

### 3.1. A Global Dataset Reveals the Continuing Spread of the mcr Gene

**Figure 1:**
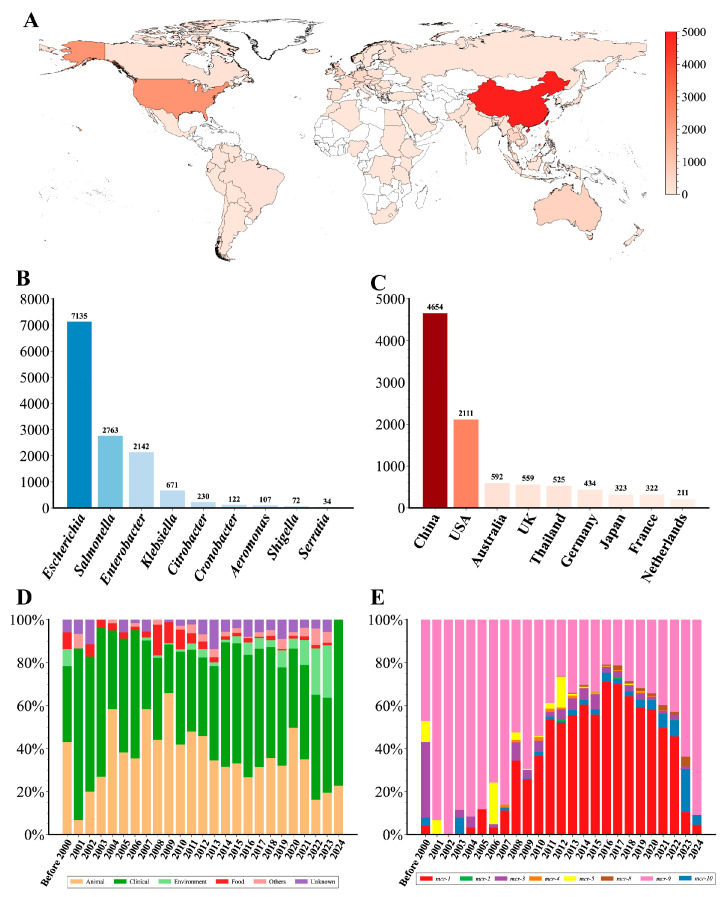
Overview of 13,344 *mcr*-harboring genomes analyzed in this study. (**A**) Global distribution of *mcr*-harboring genomes. The number of *mcr*-harboring genomes in each country is represented by the red color gradient. (**B**) Taxonomic distribution of *mcr*-harboring genomes across bacterial genera. (**C**) Top ten countries with the highest number of *mcr*-harboring genomes. (**D**) Temporal trends in the source of *mcr*-harboring genomes. The stacked bars represent the proportional distribution of the source over time. (**E**) Temporal trends of *mcr* gene variants. The stacked bars represent the relative abundance of different *mcr* variants in each year.

After quality control, we constructed a comprehensive dataset consisting of 13,344 *mcr*-positive genomes (Table S1). According to this dataset, *mcr*-positive genomes were distributed in 85 countries across 6 continents, spanning 54 species of 18 genera (Figure 1A and Table S1). *E. coli* (53.08%), *S. enterica* (20.86%), *E. hormaechei* (10.42%), and *K. pneumoniae* (4.25%) were the primary hosts for the *mcr* gene (Figure 1B). Moreover, most genomes were from China (*n* = 4654), the USA (*n* = 2111), Australia (*n* = 592), the UK (*n* = 559), and Thailand (*n* = 525) (Figure 1C). A total of 8 *mcr* variants were identified, mainly including *mcr-1* (*n* = 7417) and *mcr-9* (*n* = 4636). The proportion of *mcr-9*-positive genomes showed a declining trend prior to 2016 but gradually increased again from 2017 to 2024 (Figure 1E). Conversely, the proportion of *mcr-1*-positive genomes rose annually after 2006, reaching a peak in 2016, and then began to decline. Additionally, *mcr*-positive genomes exhibited diverse isolation sources, mainly from clinical (*n* = 6203) and animal samples (*n* = 4367) (Figure 1D). Notably, the proportion of the genomes from environment samples has shown an increasing trend in recent years.

The earliest *mcr* variant, *mcr*-3, was found in *Aeromonas salmonicida* in Norway in 1968. Over the subsequent decades, the *mcr* gene continued to disseminate across multiple genera and geographical regions, especially after 2000 (Figure S2). Further analysis showed that most *mcr* genes (70.39%, 13,862/19,693) were located on potential plasmid sequences (Table S3 and Figure S3A). Moreover, non-mobilizable, mobilizable, and conjugative plasmids contributed variably to the spread of *mcr* variants, with non-mobilizable plasmids being dominant (Figure S3B).

### 3.2. Global Spatiotemporal Transmission Characterization of Plasmid-Mediated mcr Genes

To comprehensively understand the transmission dynamics of plasmid-mediated *mcr* genes across genera or countries, we compile a dataset comprising 5,549 high-quality plasmid-derived and *mcr*-positive contigs. These contigs cover 18 genera across six continents between 1995 and 2023 (Table S2). The sequence length of these contigs ranged from 4.9 kb to 517.6 kb, with a median length of 60 kb. Among them, a total of 105 incompatibility groups were identified. The most common incompatibility groups were IncX4 (33.63%), IncI2 (28.69%), IncHI2/HI2A (18.20%), IncHI2A (2.45%), and IncP1 (2.23%). In addition, most incompatibility groups (100/105, 95.24%) only accounted for less than 2%.

**Figure 2:**
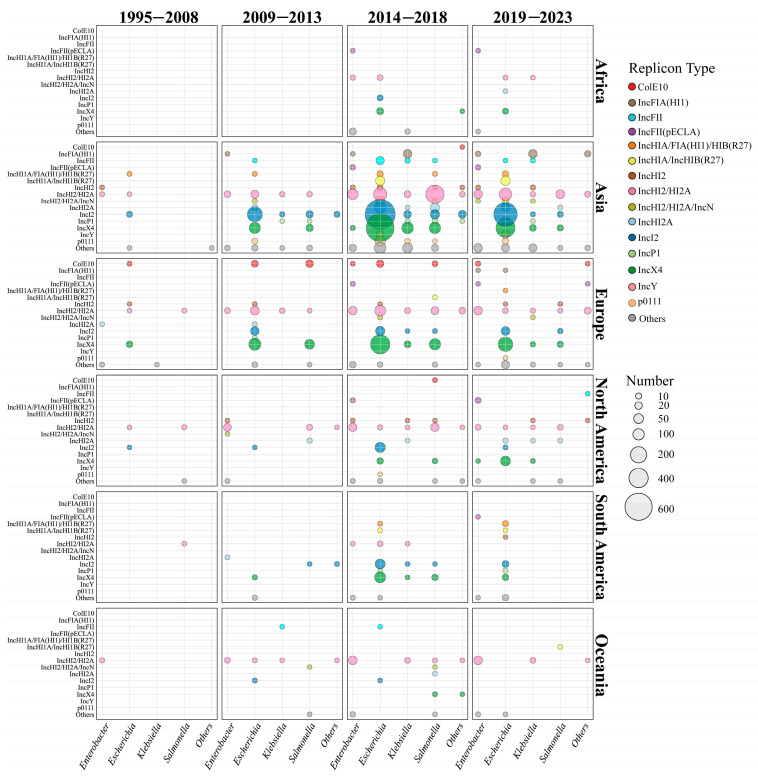
Global spatiotemporal dynamics of *mcr*-positive plasmid dissemination from 1995 to 2023. The horizontal axis represents different genera, and the vertical axis represents the top 15 plasmid types with the highest prevalence. The different plasmid types are marked in different colors, and the number of plasmids is proportional to the size of the circle.

To investigate the spatiotemporal distribution of *mcr*-carrying plasmids, we divided the time span of the dataset into four distinct periods (Figure 2). During the initial phase (1995–2008), only a small number of plasmid sequences (*n* = 43) and replicon types (*n* = 12) were identified. The first identified cross-genus transmission involved IncHI2/HI2A plasmids, which were transferred between a clinical *S. enterica* strain and an environmental *E*. *hormaechei* strain in 2000 (Figure 2). Additionally, IncX4 (2005, Spain, and animal source) and IncI2 (2006, China, and animal source) plasmids emerged during this phase but were exclusively detected within the *Escherichia* genus, without evidence of cross-genus transmission. The earliest *mcr*-carrying plasmid identified in this study was isolated from a clinical *S. enterica* strain in Belgium. The plasmid was conjugative and harbored the IncHI2/HI2A replicon. Notably, almost all the plasmid types (10/12) identified during this period re-emerged in subsequent periods, underscoring their persistence over time.

In the early transmission period (2009–2013), the number of plasmid sequences (*n* = 547) and replicon types (*n* = 28) increased gradually. At this stage, the host range of IncHI2/HI2A plasmids had extended from 3 genera to 6 genera (Figure 2 and Figure S4). In 2009, IncX4 and IncI2 plasmids began to spread from *E. coli* to other genera. As shown in Figure 2, IncI2 plasmids continued to disseminate into four other genera in addition to *Escherichia*, while IncX4 plasmids only circulated between *E. coli* and *S. enterica* (Figure 2 and Figure S4). Compared with the initial stage, the proportion of IncI2 (33.08%, 181/547) and IncX4 (32.36%, 177/547) plasmids showed a marked increase, thereby reflecting their high adaptation. Geographical analysis showed that IncI2 plasmids were mainly found in Asia (53.29%, 154/289), IncX4 plasmids were prevalent in Europe (55.33%, 109/197), and IncHI2/HI2A plasmids were predominant in North America (74.19%, 23/31) (Figure 2). Notably, broad-range IncP1 plasmids were firstly detected in a *K. pneumoniae* strain in Laos (2012 and human source), an *S. enterica* strain in China (2012 and human source), and an *E. coli* strain in the Netherlands (2013 and human source). The IncFII plasmid was firstly found in an animal sample from Japan (2012).

In the third transmission period (2014–2018), the number of plasmid sequences (*n* = 3001), replicon types (*n* = 77), and host genera (*n* = 12) increased dramatically (Figure 2 and Figure S4). IncX4 plasmids had spread from 2 genera (i.e., the *Escherichia* and *Salmonella* genera) to 6 genera. Moreover, IncX4 plasmids (30.78%, 669/2173) had disseminated rapidly in Asia, comparable to IncI2 plasmids (31.06%, 675/2173). In European regions, the proportion of IncX4 plasmid continuously increased to 64.54% (313/485). Conversely, IncHI2/HI2A plasmids showed a slightly declining trend in Asia (16.52%, 359/2173), Europe (20.41%, 99/485), and North America (38.05%, 43/113). In terms of genera, IncX4 and IncI2 plasmids were prevalent in *Escherichia* and *Salmonella*, IncHI2/HI2A predominated in *Enterobacter*, and IncX4 was dominant in *Klebsiella*. Warningly, broad-range IncP1 plasmids (*n* = 80) were epidemic in China and Southeast Asian countries, including Vietnam, Laos, Thailand, and Cambodia (Table S2), and this type of plasmid had disseminated into four genera. In addition, IncFIA(HI1) (*n* = 23) and IncY plasmids (*n* = 10) were frequently identified in *K. pneumoniae* and *E. coli* genomes from China, respectively.

In the late transmission period (2019–2023), the number of the replicon types (*n* = 66) decreased due to the declining plasmid number (*n* = 1476). IncX4, IncI2, and IncHI2/HI2A did not disseminate into new genera, although the host range of *mcr*-carrying plasmids extended to 14 genera. Overall, the plasmid dominance profiles across continents remained relatively stable. An exception was that IncX4 plasmids were predominant in North America (54.17%, 39/72) instead of IncHI2/HI2A (16.67%, 12/72). IncP1 plasmids remained spread in Southeast Asia, especially Thailand (Table S2).

The composition of plasmids varies among genera, and even within a genus, the prevalence varies by region. Our further analysis indicated that geographic distribution, the genus, and *mcr* variants significantly influence the composition of plasmid types (Figure S5).

### 3.3. Intra-Escherichia coli and Salmonella enterica Dissemination by Clonal Expansion

Given the dominated host role of *E. coli* and *S. enterica* in the transmission of the *mcr* gene, a more in-depth analysis was constructed. Based on 13,344 genomes, MLST analysis revealed a remarkable diversity among *mcr*-positive *E. coli* (*n* = 7083) and *S. enterica* (*n* = 2763) genomes, which were assigned to 842 and 115 unique STs, respectively (Table S1 and Figure S6). Of them, ST10 and ST34 were the predominant in *E. coli* (616/7083, 8.70%) and *S. enterica* (938/2763, 33.95%) genomes, respectively. Among the STs that contained >10 genomes, 14 STs (12.73%, 14/110) in *E. coli* and 13 STs (43.33%, 13/30) in *S. enterica* were highly concentrated in a single country (with the proportions exceeding 80%).

To investigate the transmission patterns of the plasmid-mediated *mcr* gene within epidemic clonal lineages, we selected the ST10 *E. coli* (*n* = 346) and ST34 *S. enterica* (*n* = 248) for detailed analysis, respectively (Table S2). We extracted their core genome and categorized them into 37 and 41 distinct groups, respectively, based on a core genome single-nucleotide polymorphism (SNP) threshold of 20 (Supplementary Materials: Tables S4 and S5). We found that most of the groups contained only one type of plasmid (Figure S7) and were isolated from a single host or country (Figure S8), thereby highlighting the role of clonal transmission and clinical outbreak in *mcr* dissemination. Moreover, within-plasmid-type SNP distances were significantly lower than between-plasmid-type SNP distances in ST10 *E. coli* and ST34 *S. enterica* (*p* < 0.05).

### 3.4. Transmission Dynamics Between STs in Escherichia coli and Salmonella enterica in Four Periods

A total of 387 genetic environments were identified in this study, with “*mcr-1*-*pap2*” (56.10%, 3113/5549) and “IS*Apal1*-*mcr-1*-*pap2*” (9.91%, 550/5549) being predominant (Table S2). IS*Apal1*, IS*26*, IS*903*, IS*Kpn40*, Tn*As2*, and Tn*3* were detected in *mcr*-related genetic environments. To better speculate the transmission events of *mcr*, we combined *mcr* variants, the secondary unit identified by Mob-suite with its associated genetic environments, to form a new variable referred to as the transmission unit. The plasmid-borne contigs were regarded as having potential epidemiological association if they shared the same transmission unit. We investigated the potential horizontal dissemination events between STs during the four periods mentioned above in *E. coli* and *S. enterica* (Supplementary Materials: Tables S6 and S7). A total of 43 and 10 transmission units were involved in cross-ST transmission in *E. coli* and *S. enterica*, respectively.

In the initiate stage (1995–2008), only 3 transmission units linked to inter-ST transmission were identified in *E. coli*, and all the transmission units reappeared in the subsequent periods, indicating their stability and persistence (Figure S9A). However, there were no cross-ST dissemination events in *S. enterica.*

In the early transmission period (2009–2013), 14 transmission units linked to inter-ST transmission were identified in *E. coli* (Figure S9B). Notably, AI859*mcr-1*_type1 (932/2025, 46.0%) and AI350*mcr-1*_type1 (826/2025, 40.8%) accounted for most cross-ST transmission events, which contained IncX4 and IncI2 replicons, respectively. There were only 10 STs shared between AI859*mcr-1*_type1- (spanning 44 STs) and AI350*mcr-1*_type1-related transmission events (spanning 43 STs). The transmission units AJ047*mcr-1*_type19 and AO510*mcr-5*_type1 only appeared once, which was indicative of transient transmission events. Regarding *S. enterica*, there were 4 transmission units related to cross-ST transmission, respectively (Figure S10A).

**Figure 3:**
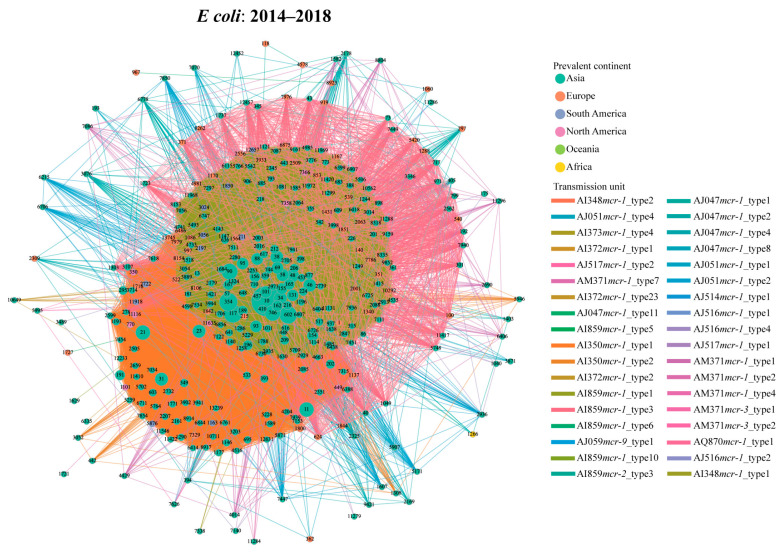
Transmission network of *mcr*-harboring plasmid across *E. coli* STs during the peak period (2014–2018) based on transmission units. The node represents the *E. coli* ST, and the edge indicates potential transmission event. The node color corresponds to the geographical regions where each ST is most prevalent, and the edge color corresponds to the type of the transmission unit.

During the third period (2014–2018), the number of the transmission units reached the peak, with 34 transmission units carrying 15 genetic environments in *E. coli* (Figure 3). Compared with the early stage, the proportion of AI859*mcr-1*_type1-related transmission events (14,091/27,636, 51.0%) increased slightly, whereas the prevalence of the AI350*mcr-1*_type1 (9492/27,636, 34.3%) showed a declining trend. There was no obvious increase for the proportion of shared STs (112 STs) between AI859*mcr-1*_type1- (spanning 346 STs) and AI350*mcr-1*_type1-related transmission events (spanning 297 STs). Despite numerous potential dissemination events, the selectivity of their host STs resulted in a geographical limitation, predominantly distributed within Asia (Figure 3). Notably, AM371*mcr-1*_type1-associated transmission events (harbored by IncP1 plasmids) occurred 215 times, spanning across 19 STs (Table S2). Regarding *S. enterica*, 8 transmission units have been found to be associated with cross-ST transfer (Figure S10B). AI859*mcr-1*_type3 (90/182, 49.5%) exhibited high frequency among all possible events in *S. enterica*. Interestingly, each instance of this unit contains an IncX4 plasmid.

In the late transmission period (2019–2023), the number of the transmission units (*n* = 23) and genetic environments (*n* = 11) decreased in *E. coli* (Figure S9C). Notably, 5 transmission units and 5 genetic environments identified during this period had not been documented in previous periods. Nevertheless, the primary transmission units responsible for *mcr* transmission remained largely unchanged. As for *S. enterica*, AJ051*mcr-1*_type1-realted transmission events (harbored by the IncHI2A plasmid) firstly occurred within this species, which spanned across three distinct STs (i.e., ST17, ST34, and ST155).

### 3.5. Broad-Range IncP1 Plasmids Are Important Carriers for Cross-Genus and Cross-Region Transmission of the mcr Gene

According to the results of the transmission unit, 79 transmission units were involved in cross-genus transmission. Among them, 35 transmission units spanned across three or more than three genera, and 11 transmission units spanned across four or more than four genera (Figure S11). Notably, the AJ059*mcr-9*_type1 transmission units (characterized by the genetic environment of “IS*903B*-*mcr-9*-*wbuc*-IS*26*” and harbored by the IncHI2/HI2A plasmid) were distributed in 11 genera. For these cross-genus transmission units, the most common plasmid types were IncHI2/HI2A (43.04%, 34/79), IncX4 (8.86%, 7/79), and IncI2 plasmid (6.33%, 5/79). Further analysis found that more than one-third of transmission units (36.71%, 29/79) were related to cross-ST transmission in *E. coli* and *S. enterica*.

**Figure 4:**
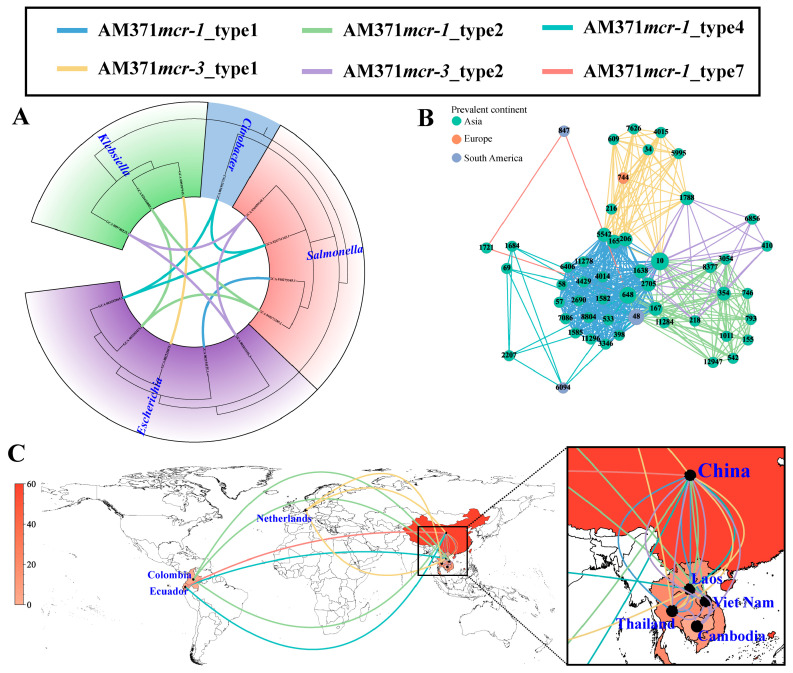
Cross-genus, cross-ST, and cross-region transmission of IncP1 plasmids based on transmission units. (**A**) Cross-genus transmission network of IncP1 plasmids. The phylogenetic tree is constructed based on 120 conserved marker genes extracted by GTDB-TK v2.6.1 software. The connecting line between tree tips indicates putative transmission events, with line colors representing different transmission units. (**B**) Cross-ST transmission network of IncP1 plasmid in *E. coli*. The node represents the *E. coli* ST, and the edge indicates a potential transmission event. (**C**) Cross-region transmission network of IncP1 plasmid in *E. coli*. The number of IncP1 plasmids in each country is represented by the red color gradient. The connecting line represents potential transmission events.

In addition to these prevalent plasmid types, broad-range IncP1 plasmids displayed a strong association with cross-genus transmission, comprising five distinct transmission units (6.32%, 5/79) (Figure 4A). Of these, three transmission units (i.e., AM371*mcr-1*_type2, AM371*mcr-1*_type4, and AM371*mcr-3*_type2) spanned across three different genera, and the remaining two transmission units (i.e., AM371*mcr-1*_type1 and AM371*mcr-3*_type1) spanned two genera. However, all observed cross-genus transmission events were confined to the *Enterobacteriaceae* family. The five transmission units also served as the medium of cross-ST transmission in *E. coli*, spanning 51 distinct STs (Figure 4B). AM371*mcr-1*_type1 and AM371*mcr-1*_type2 dominated IncP1-mediated cross-ST transmission, accounting for 65.92% (176/267) and 15.73% (42/267) of such events, respectively. The two transmission units were characterized by the genetic environment of “*mcr-1*-*pap2*” and “IS*Apl1*-*mcr-1*-*pap2*”, respectively (Figure S12). Moreover, they can be traced back to a clinical setting in China in 2012, with their initial isolation from an *S. enterica* ST34 and an *E. coli* ST793 strain, respectively. Notably, AM371*mcr-1*_type1 and AM371*mcr-3*_type2 only circulated in Southeast Asia (Figure 4C).

### 3.6. Transmission History of IncX4 Plasmid Based on Bayesian Analysis

**Figure 5:**
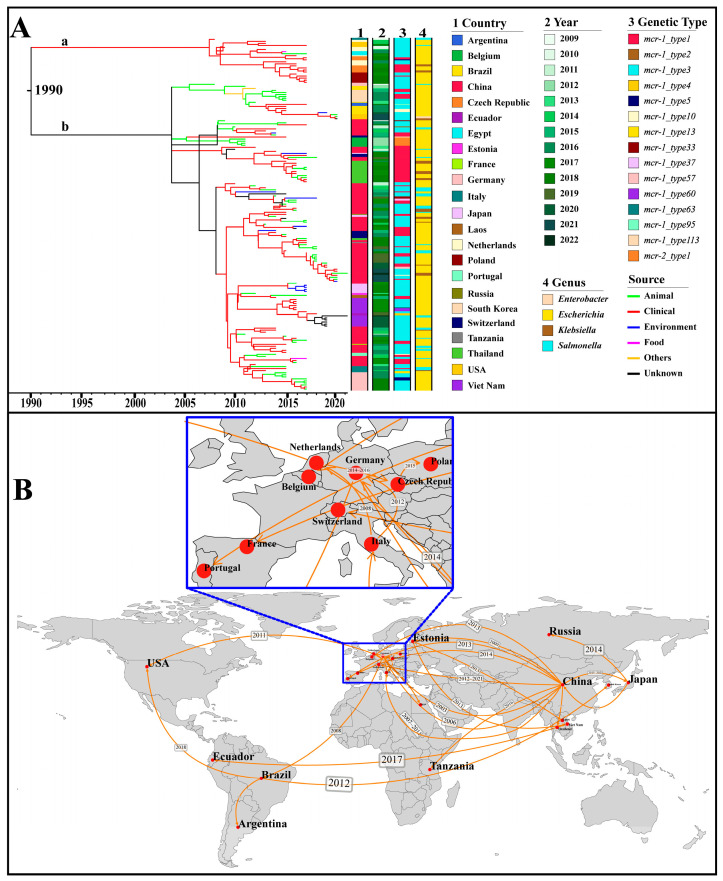
Phylogenetic analysis based on the core genes of 173 IncX4 plasmids with the assembly level of “complete”. (**A**) The Maximum Clade Credibility Tree (MCC Tree). The horizontal axis represents presumed divergence time. The heat map indicates country, collection year, genetic type (i.e., genetic environment), and genus. The branches are colored by the sources. The group “a” and “b” are marked in their corresponding position. (**B**) Geographical transmission of the IncX4 plasmid across countries. The geographic spread of the IncX4 plasmids is inferred through ancestral state reconstruction of the timed phylogenetic tree. The transmission time of IncX4 plasmids between countries is marked in the corresponding position.

The IncX4 plasmids, which accounted for the highest number among all Inc types, were used as representatives for the analyses of origin and transmission routes. As shown in Figure 5A, IncX4-type plasmids were speculated to have originated in 1990 and mainly spread in two main groups, a and b. The plasmids of group a were distributed in geographical regions outside China. Source analysis showed that group b emerged from an animal source and gradually transmitted to humans and the environment. By utilizing the country of sampling as a discrete trait, we performed a phylogeographic analysis to reconstruct possible transmission routes (Figure 5B). The IncX4 plasmid could be traced back to China and subsequently expanded predominantly to other countries. In the early 21st century, IncX4 plasmids spread from China to Germany, Italy, and the Netherlands, leading to outbreaks in European countries. Subsequently, from 2010, they continued to expand across European countries and were introduced from China to Thailand, from Brazil to South Korea, and from the Netherlands to the USA (Figure 5B).

A total of thirteen genetic environments were identified in the 173 IncX4 plasmids. Among them, *mcr-1*_type3 (105/173, 60.69%) and *mcr-1*_type1 (48/173, 27.75%) were the most prevalent. Notably, *mcr-1*_type3 was the earliest discovered genetic environment, characterized by the structure of “IS*26*-*hp*-*hp*-*hp*-*hp*-*mcr-1*-*pap2*”. Subsequently, a new insertion sequence, IS*1294*, was integrated into the downstream region of *mcr-1*, forming a new genetic environment known as *mcr-1*_type33. Next, *mcr-1*_type1 was identified, carrying the core structure of “*mcr-1*-*pap2*” without any IS elements (Figure 5 and Table S2).

## 4. Discussion

The emergence and dissemination of colistin resistance pose a critical challenge in combating MDR Gram-negative bacterial infections, particularly carbapenem-resistant strains [7,29]. In 2015, a surveillance study from China identified the emergence of the plasmid-borne *mcr-1* gene in *E. coli*, the first known mobile colistin resistance determinant [13]. Alarmingly, within just three months of its initial report, *mcr* transmission were detected across more than twenty countries [30,31]. Through large-scale genomic screening of over 10,000 bacterial genomes, we identified *mcr* genes across 85 countries and more than 50 bacterial species, expanding current understanding of their global epidemiology. In this study, the first *mcr* variant (i.e., *mcr-3*) was discovered in an *Aeromonas salmonicida* isolate collected in Norway in 1968, further supporting the hypothesis that *Aeromonas* species serve as an ancestral reservoir of *mcr-3* evolution [32]. Furthermore, temporal analysis displayed accelerated transmission of *mcr* variants after 2000. This trend coincides with a reported 13% increase in global polymyxin consumption between 2000 and 2010, reinforcing escalating antimicrobial use as a driving force behind *mcr* dissemination and evolution [7]. Although regulatory measures, including agricultural colistin bans and reduced clinical usage, have helped decrease *mcr* prevalence, ongoing surveillance continues to document frequent *mcr* dissemination in humans, animals, and environments [33,34,35]. Consistent with these findings, we observed the continuing cross-region and cross-genera spread of the *mcr* gene within the “One health” continuum. Considering the contribution of plasmids to *mcr* spread, tracking transmission dynamics of *mcr*-carrying plasmids is essential for informing infection control strategies and antimicrobial stewardship efforts.

It has been thought that *mcr-1* is the most common *mcr* variant [17,36]. Moreover, several surveillance studies demonstrated its detection frequency increased between 2011 and 2017 after sporadic identification in the 1980s, followed by the decline in recent years [13,33]. Similarly, we observed that the proportion of *mcr-1*-positive genomes peaked in 2016. The observed increase in *mcr-1* detection rates may be attributed, in part, to heightened scientific focus on this resistance determinant. Nevertheless, a metagenomic analysis revealed that *mcr-9* exhibited a broader geographical distribution than *mcr-1* [37]. Previous investigation showed that *mcr-9* confers weaker colistin resistance than *mcr-1*, exhibiting negligible baseline expression without colistin induction [38,39]. Characterized by low-level colistin resistance, *mcr-9* appears to have exploited this phenotypically subtle feature to facilitate persistent colonization and covert dissemination, evading conventional detection methods [40].

By integrating genome assemblies from three databases, we identified 105 distinct plasmid replicon types in *mcr*-positive plasmids, underscoring their extensive, previously uncharacterized genetic diversity. Epidemiological data consistently demonstrated that IncX4, IncI2, and IncHI2/HI2A plasmids predominantly drive the spread of the *mcr* gene [14,36]. According to our screening results, IncHI2/HI2A plasmids were initially predominant, with IncI2 and IncX4 plasmids becoming more prevalent recently. It has been thought that the decline of *mcr-1* prevalence following the ban on agricultural colistin can be attributed to the fitness burden imposed by *mcr-1* [41,42]. Indeed, the overexpression of the *mcr* gene can decrease bacterial growth rate and disrupt cell membrane structure, potentially limiting its persistence in bacterial populations [41]. Nevertheless, the low copy number and high conjugation transfer ability of IncX4 and IncI2 plasmids could compensate for the fitness cost, which partly explains their continued widespread prevalence in the absence of antibiotic pressure [43,44].

Compared to IncI2 and IncHI2/HI2A plasmids, IncX4 plasmids are characterized by high transferability, which often encodes a Type IV secretion system essential for conjugation transfer across cells [45]. In vitro experiments have demonstrated that IncX4 plasmids could transfer the *mcr-1* gene from *E. coli*, *K. pneumoniae*, and *P. aeruginosa* to laboratory recipient isolates [46,47,48]. In the current study, IncX4 plasmids have become the most common and evolutionarily successful carriers for *mcr* genes. Phylogeography reconstruction showed that the rapid expansion of the IncX4 plasmid occurred after 2010, spreading from China to Europe and other regions. Moreover, group b in IncX4 plasmids originated from an animal source, thereby providing direct evidence for zoonotic transmission for antibiotic resistance. This underscores the critical importance of the One Health framework for controlling the spread of resistance genes across human, animal, and environmental boundaries [49]. By further mapping global dissemination of *mcr*-positive plasmids, we observed that the three plasmid types (i.e., IncX4, IncI2, and IncHI2/HI2A) have now stabilized in both geographic distribution and host range. In addition to these narrow-host-range plasmids, there were increasing reports about IncP1 plasmids carrying the *mcr-1* or *mcr-3* gene [50,51]. We found that these broad-host-range IncP1 plasmids were mainly circulated in China and Southeast Asia, with sporadic occurrences in Europe and South America. According to the analysis results of transmission units, IncP1 has achieved cross-continent and cross-species transmission, even cross-ST spread within *E. coli*, calling for enhanced surveillance for this plasmid lineage.

The dissemination of insertion sequences (ISs) or transposons between different plasmid lineages represents an additional mechanism facilitating HGT of ARGs [52]. It has been proposed that *mcr-1* could be mobilized by an IS*Apl1*-related composite transposon [53]. In some cases, the transposon has lost one or both copies of IS*Apl1*, suggesting a loss of mobility [54]. Here, we observed that the majority of *mcr-1*-related genetic environments lacked IS*Apl1* elements (e.g., *mcr-1*_type1), which may contribute to the stable maintenance of *mcr-1* across various genomic backgrounds. IS*26* is the second most common insertion sequence for *mcr-1* [36]. IS*26* could mediate transposon truncation (e.g., Tn*2* upstream of *bla*_TEM-1_), plasmid fusion, and genomic recombination under antimicrobial selection pressure [55,56]. Previous investigation showed IS*26* mediated the cointegration of an IncN1-F33:A:B- plasmid and an *mcr-1*-haboring phage-like plasmid, with a frequency of 1.75 × 10^−4^ [52]. Regarding the *mcr-9* gene, we found that IS*903* was frequently identified upstream of *mcr-9*, while IS*1R* and IS*26* were commonly detected downstream. *mcr-3* was strongly associated with Tn*As2*, IS*Kpn40*, and IS*26*. The variation in IS association across *mcr* variants underscores their distinct evolutionary and dissemination pathways.

In addition to the well-documented contribution of plasmid-mediated horizontal gene transfer to ARGs spread, clonal transmission represents another important mechanism driving resistance propagation [51]. Accumulating epidemiological evidence has established that the global dissemination of *bla*_CTX-M-15_ and *bla*_KPC-2_ genes is largely attributed to clonal expansion of internationally successful high-risk clones, specifically *E. coli* ST131 and *K. pneumoniae* ST258/11, respectively [57,58]. Likely, large-scale genomic analysis of over one thousand *mcr*-harboring *K. pneumoniae* genomes has provided evidence for the role of clonal transmission in *mcr* spread [19]. Here, we observed that most genomes harboring < 20 SNPs difference carried conserved plasmid lineage, supporting the importance of clonal transmission and clinical outbreak in *mcr* dissemination. However, compared to *bla*_KPC-2_ and *bla*_CTX-M-15_, clonal transmission appears to play a less dominant role in *mcr* spread. Previous genomic analysis showed that ST11/ST258 clones represented over 70% of *bla*_KPC-2_-positive *K. pneumoniae* genomes, and ST131 accounted for more than 60% of *bla*_CTX-M-15_-positive *E. coli* genomes [58,59]. Regarding the *mcr* gene, the predominant STs only account for less than 40% of cases in their respective species, suggesting that HGT serves as the primary driver of *mcr* dissemination rather than clonal spread, a pattern similar to *bla*_NDM-1_ [60]. Therefore, tailored control strategies should be implemented based on the predominant transmission mechanisms of specific resistance genes to optimize containment efforts.

Notably, some limitations should be acknowledged. Firstly, there is a sampling bias. The disproportionate focus on colistin usage in veterinary and clinical settings has led to an overrepresentation of these samples in public databases compared to environmental isolates. Moreover, the disparities in sequencing technology development may introduce geographic and temporal biases in publicly available genomic data. Moving toward a more systematic, balanced, and representative sampling framework across all reservoirs (clinical, veterinary, environmental) will be crucial for obtaining a scientifically robust epidemiological perspective. This will enable a more accurate understanding of the true dissemination dynamics and evolutionary pathways of *mcr*-carrying plasmids. Secondly, existing plasmid classification systems require refinement to better resolve the full spectrum of plasmid diversity. Thirdly, frequent recombination events complicate phylogeography analyses, underscoring the urgent need for innovative computational frameworks to accurately trace plasmid evolution.

## 5. Conclusions

In summary, we tracked the transmission dynamics of plasmid-mediated *mcr* genes by comprehensive genome and plasmid mining. The dissemination of the dominated plasmid lineages (i.e., IncX4, IncI2, and IncHI2/HI2A) across regions and hosts has entered a stable phase through HGT and clonal expansion, driven primarily by HGT, with clonal expansion also contributing. Our findings fundamentally advance current understanding of *mcr* spread and underscore the need for developing targeted interventions to interrupt ARGs transmission mediated by HGT.

## Data Availability

The data presented in this study are publicly available in NCBI Pathogen Detection database (https://www.ncbi.nlm.nih.gov/pathogens/, accessed on 30 April 2024), NCBI RefSeq database (ftp://ftp.ncbi.nlm.nih.gov/refseq/release/plasmid/, accessed on 30 April 2024), and PLSDB database (https://ccb-microbe.cs.uni-saarland.de/plsdb, accessed on 30 April 2024). The original contributions presented in this study are included in the article/Supplementary Material.

## Supplementary Materials

The following supporting information can be downloaded at: https://www.mdpi.com/article/10.3390/microorganisms14010028/s1. Figure S1. Flowchart of the whole analysis used in this study. Figure S2. Chronological discovery of the *mcr* variants in each genus (A) and country (B) based on 13344 *mcr*-harboring genomes. The first isolation time of *mcr* variant in each country and genus was marked, and different *mcr* variants are represented by different colors. Figure S3. Temporal trends of *mcr* gene localization over time. (A) Annual proportion of *mcr* genes detected in chromosome (blue color) versus plasmid (red color). (B) Temporal distribution of plasmid-mediated *mcr* gene among conjugative, mobilizable, and non-conjugative plasmids. Figure S4. Global spatiotemporal dynamics of *mcr*-harboring plasmid dissemination across uncommon genera. The horizontal axis represents collection year, and the vertical axis represents the genus. The different plasmid types are marked in different colors, and the number of plasmids is proportional to the size of the circle. Figure S5. Distribution of plasmid replicon types across continents (A), *mcr* variants (B), and genus (C). Different plasmid replicons are marked in different colors. Figure S6. Minimum spanning trees of *mcr*-harboring *E. coli* (A) and *S. enterica* genomes (B) based on MLST results. Each node corresponds to a distinct ST, with node color representing different countries. Figure S7. Heatmap of plasmid prevalence in each SNP cluster group of ST10 *E. coli* and ST34 *S. enterica.* Figure S8. Distribution of each SNP cluster group of ST10 *E. coli* (A) and ST34 *S. enterica* (B) across sources and countries. Figure S9. Transmission network of *mcr*-harboring plasmid across *E. coli* STs in the three periods: (A)1995-2008, (B)2009-2013, and (C)2019-2023. Figure S10. Transmission network of *mcr*-harboring plasmid across *S. enterica* STs from 2009 to 2023. Figure S11. Cross-genus transmission network of *mcr*-harboring plasmids. The phylogenetic tree is constructed based on 120 conserved marker genes extracted by GTDB-TK software. The connecting line between tree tips indicates putative transmission events, with line colors representing different transmission units and line styles indicating transmission range (solid lines: <4 genera; dashed lines: ≥4 genera). The transmission units spanning ≥ 4 genera are marked at the top-left position. Figure S12. Genetic environment of *mcr*-flanking genes in IncP1 plasmids. The different genes are represented by different colors, and the gene name is marked at the top position. The transcription direction of the gene is represented by the arrow. Table S1. Basic information of 13,344 genomes used in this study. Table S2. Basic information of 5549 genomes used in this study. Table S3. Basic information of 19,693 mcr-positive contigs extracted from 13344 genomes. Table S4. The clustering results of ST10 Escherichia coli. Table S5. The clustering results of ST34 Salmonella enterica. Table S6. Basic information of cross-ST transmission units varied in 4 distinct periods in Escherichia coli. Table S7. Basic information of cross-ST transmission units varied in 4 distinct periods in Salmonella enterica.
