# Supplementary material for: Tracking Global Transmission Dynamics of the Plasmid-Mediated mcr Gene: A Genomic Epidemiological Analysis
Source: Microorganisms. 2025 Dec 22;14(1):28. doi: 10.3390/microorganisms14010028 (PMC12844032; doi:10.3390/microorganisms14010028)
Supplement: Supplementary file 1 [file microorganisms-14-00028-s001.zip › microorganisms-3998395-supplementary.pdf]

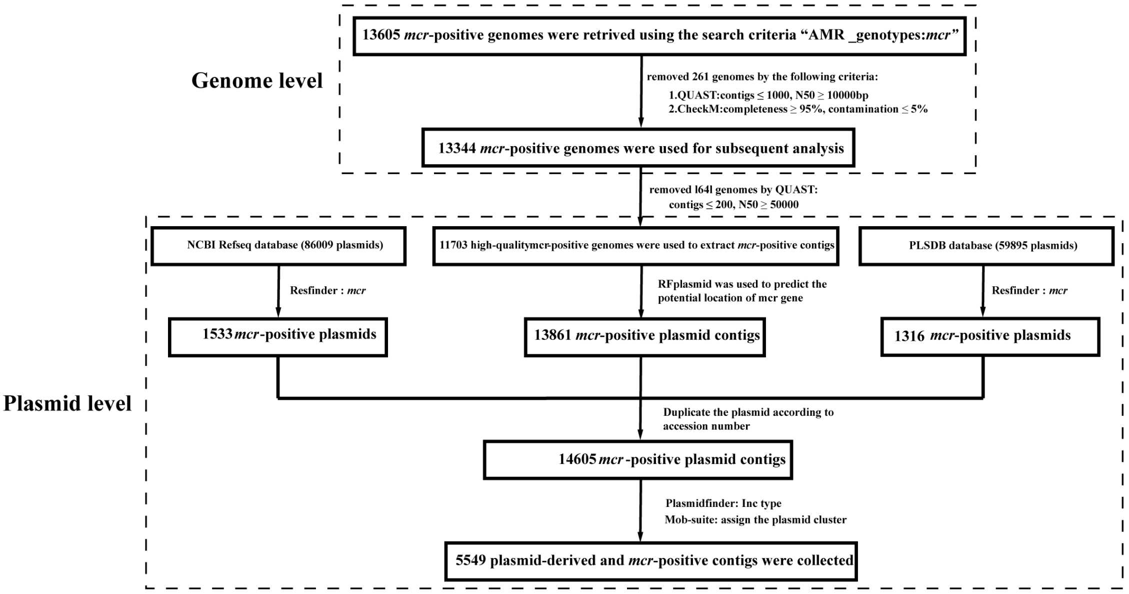

Figure S1. Flowchart of the whole analysis used in this study. Firstly, 11703 high-quality genomes were selected from 13605 *mcr*-positive genomes identified in the NCBI Pathogen Detection database (number of contigs ≤ 200 and N50 length ≥ 50000bp). Subsequently, *mcr*-positive contigs were extracted from these high-quality genomes by running ResFinder v.4.1. To determine whether the *mcr* gene is located on the chromosomes or plasmids, we applied RFplasmid v0.0.18) to predict their potential location. Additionally, we downloaded 86009 and 59895 plasmids with "complete" assembly level from the NCBI RefSeq and PLSDB plasmid databases (access date: April 30, 2024), respectively. Similarly, ResFinder v.4.1 was employed to identify *mcr*-positive plasmids with default parameters. In cases where genomes of the same strain were redundantly recorded across these databases, the genome with the lowest number of contigs and the highest genome sequencing coverage was selected. Besides, PlasmidFinder v.2.2.1 was utilized to identify plasmid replicons, and Mob-suite v.3.19 was applied to assign the plasmid cluster and predict the plasmid mobility. Ultimately, a total of 5549 putative plasmid-derived contigs were included for final plasmid analysis (contained at least one plasmid replicon and assigned a plasmid cluster ID) (Table S3).

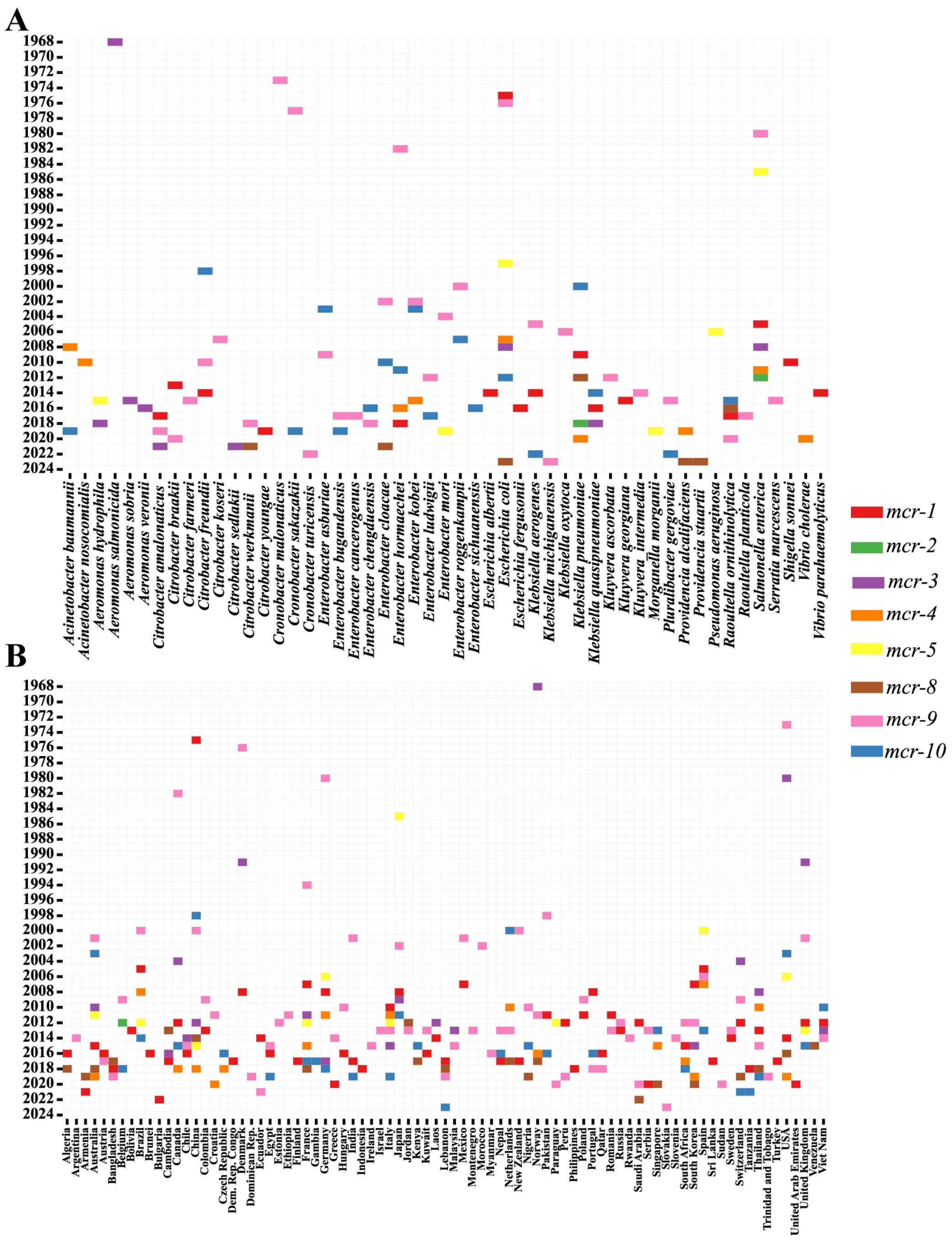

Figure S2. Chronological discovery of *mcr* variants in each genus (A) and country (B) based on 13344 *mcr*-harboring genomes. The first isolation time of the *mcr* variant in each country and genus was marked, and different *mcr* variants are represented by different colors.

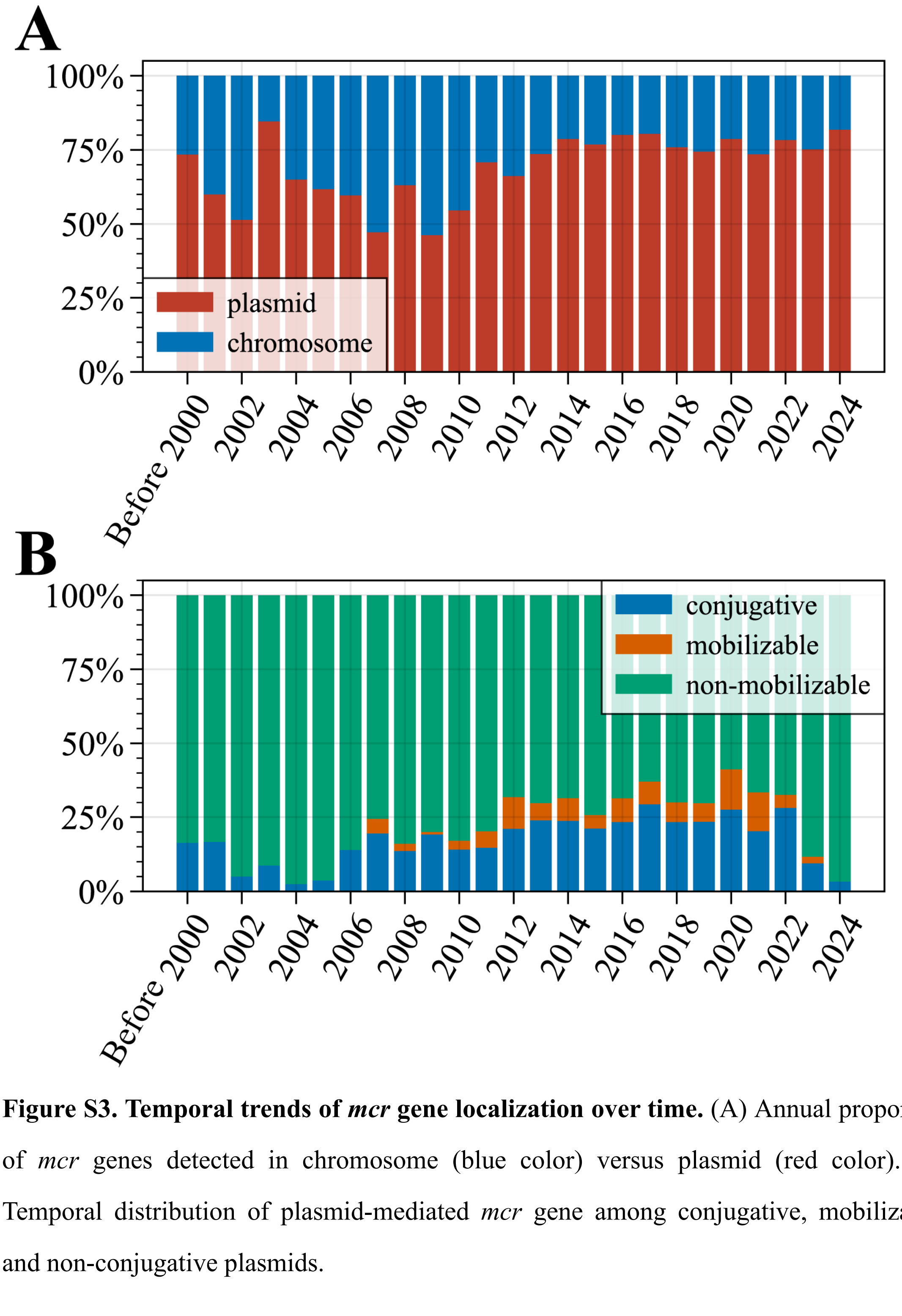

Figure S3. Temporal trends of *mcr* gene localization over time. (A) Annual proportion of *mcr* genes detected in chromosome (blue color) versus plasmid (red color). (B) Temporal distribution of plasmid-mediated *mcr* gene among conjugative, mobilizable, and non-conjugative plasmids.

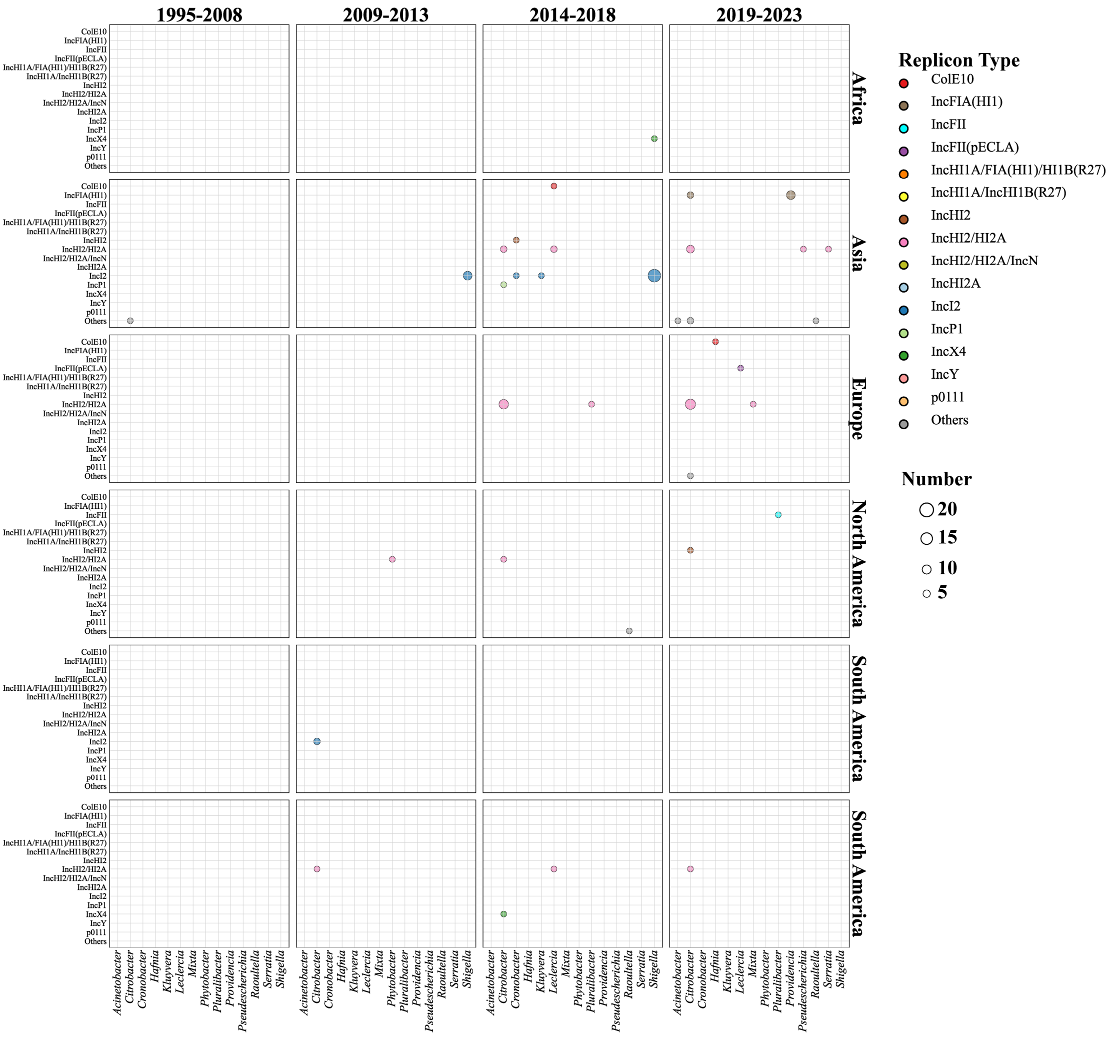

Figure S4. Global spatiotemporal dynamics of *mcr*-harboring plasmid dissemination across uncommon genera. The horizontal axis represents collection year, and the vertical axis represents the genus. The different plasmid types are marked in different colors, and the number of plasmids is proportional to the size of the circle.

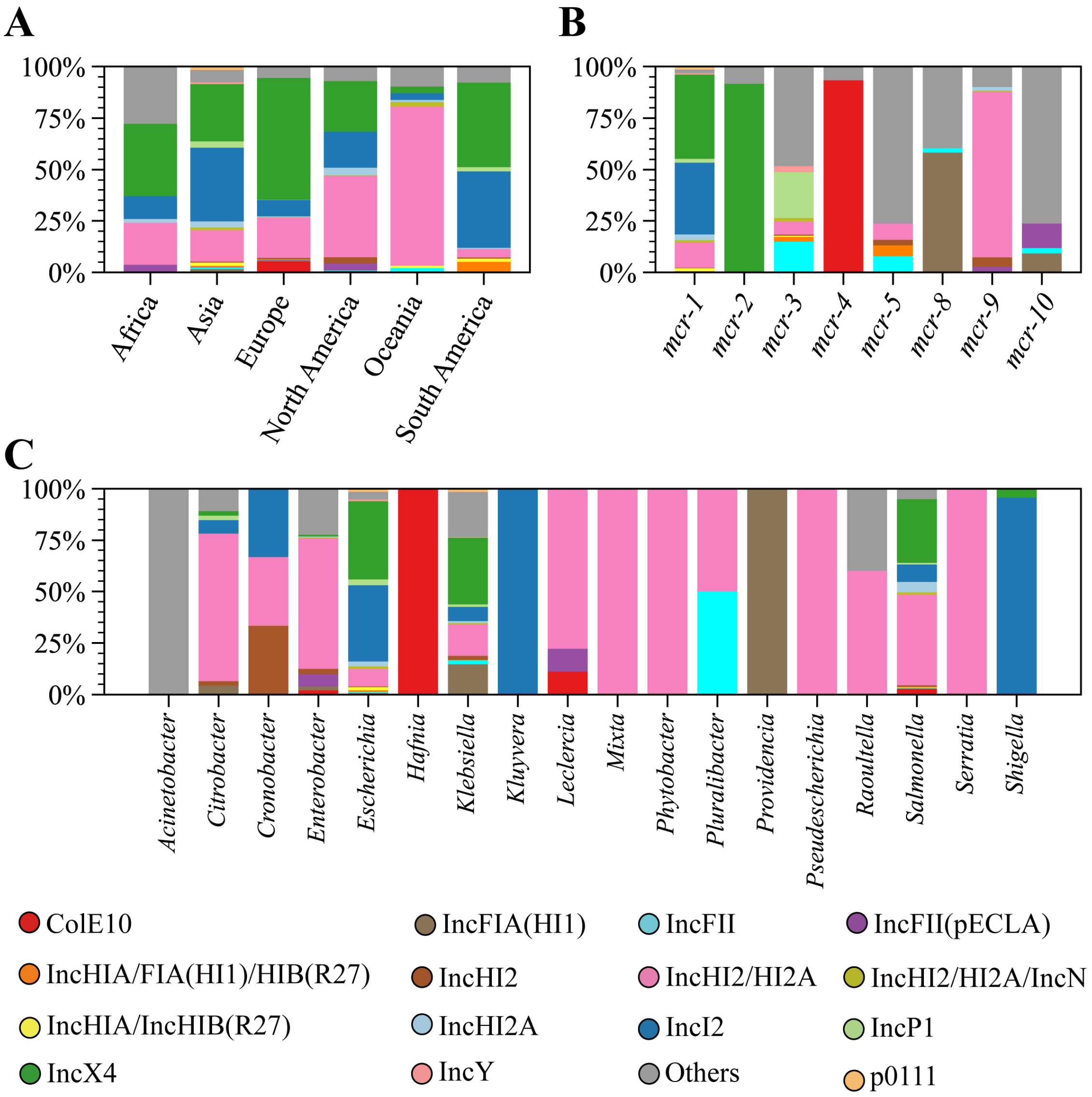

Figure S5. Distribution of plasmid replicon types across continents (A), *mcr* variants (B), and genus (C). Different plasmid replicons are marked in different colors.

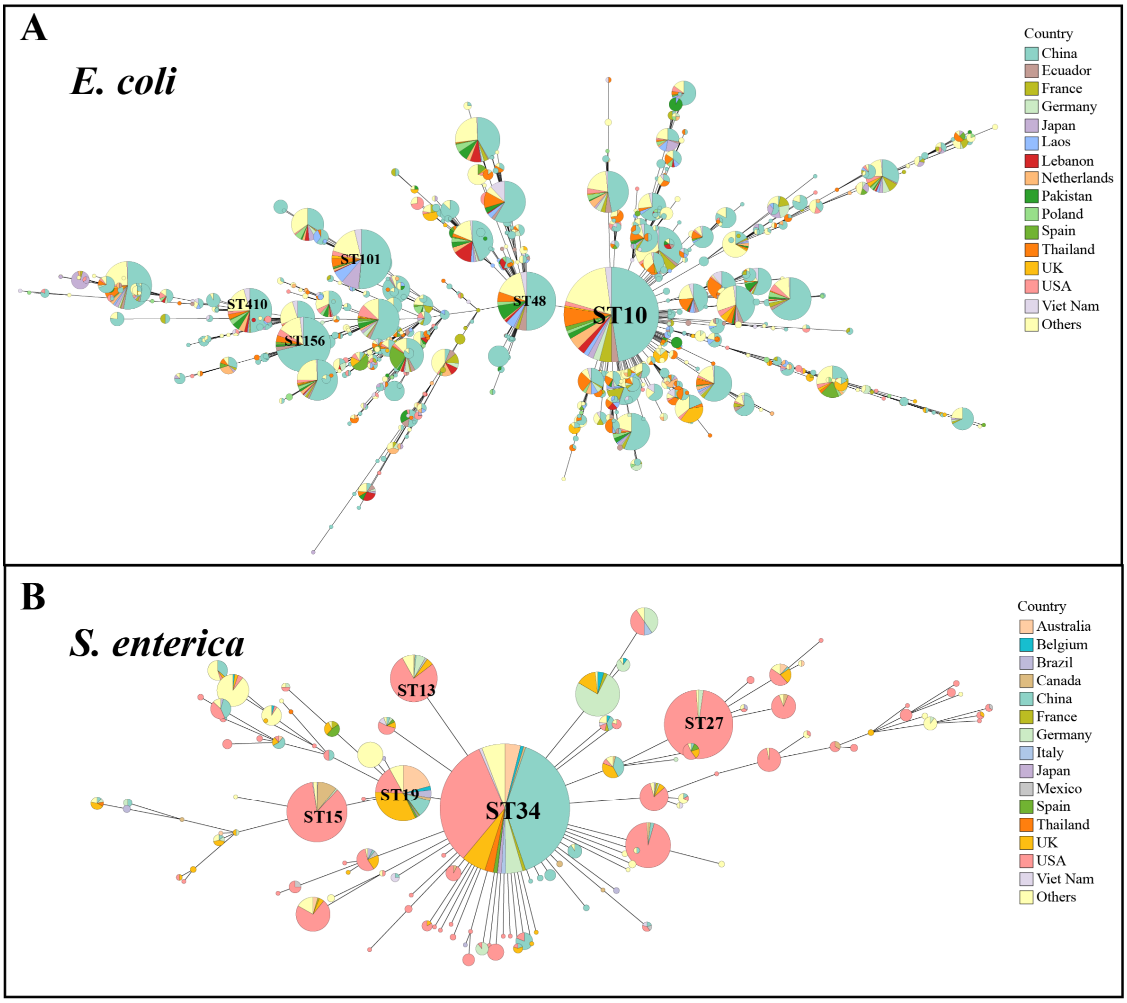

Figure S6. Minimum spanning trees of *mcr*-harboring *E. coli* (A) and *S. enterica* genome (B) based on MLST results. Each node corresponds to a distinct ST, with node color representing different countries.

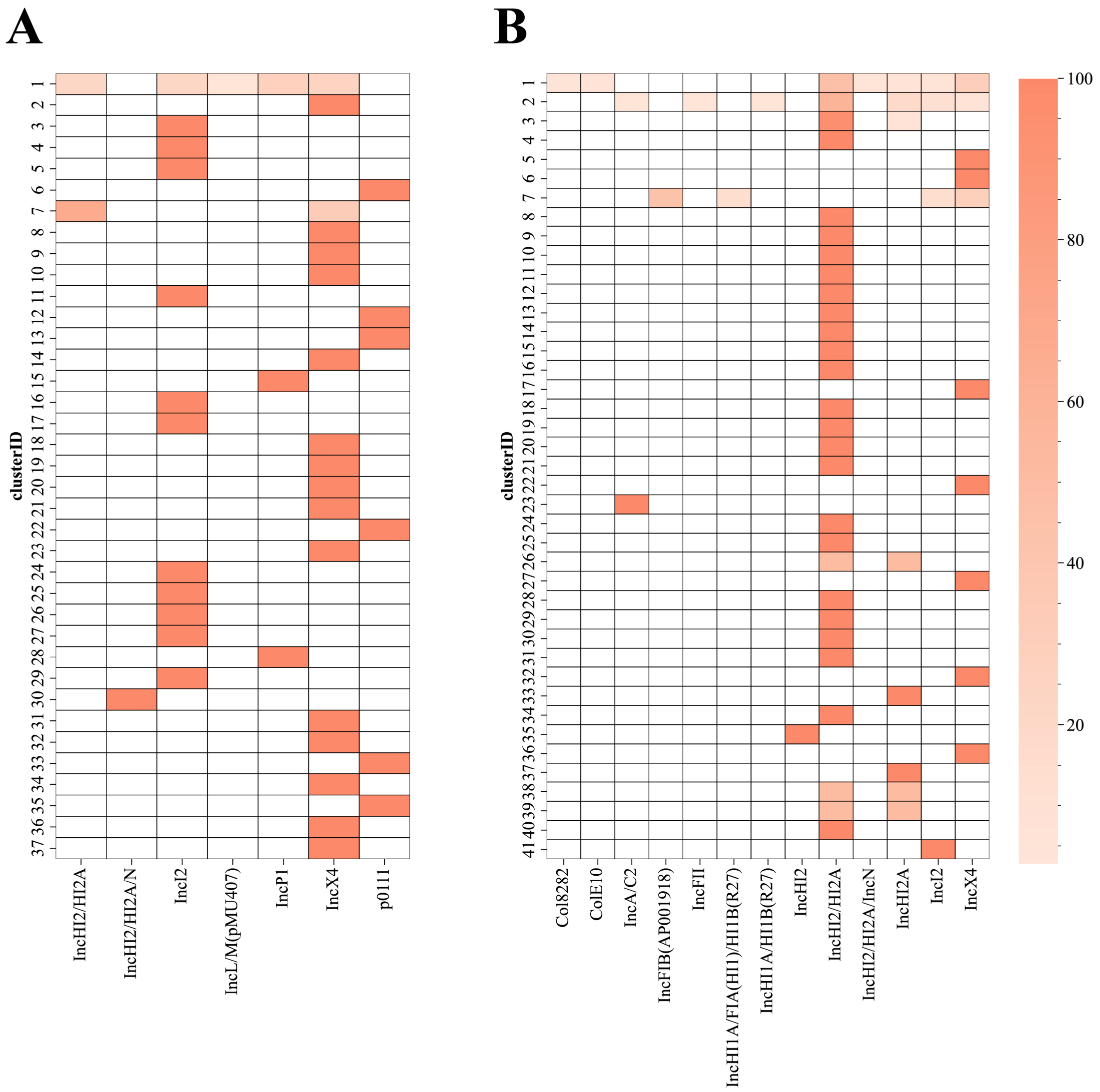

Figure S7. Heatmap of plasmid prevalence in each SNP cluster group of ST10 *E. coli* and ST34 *S. enterica*. Singleton clusters are not shown.

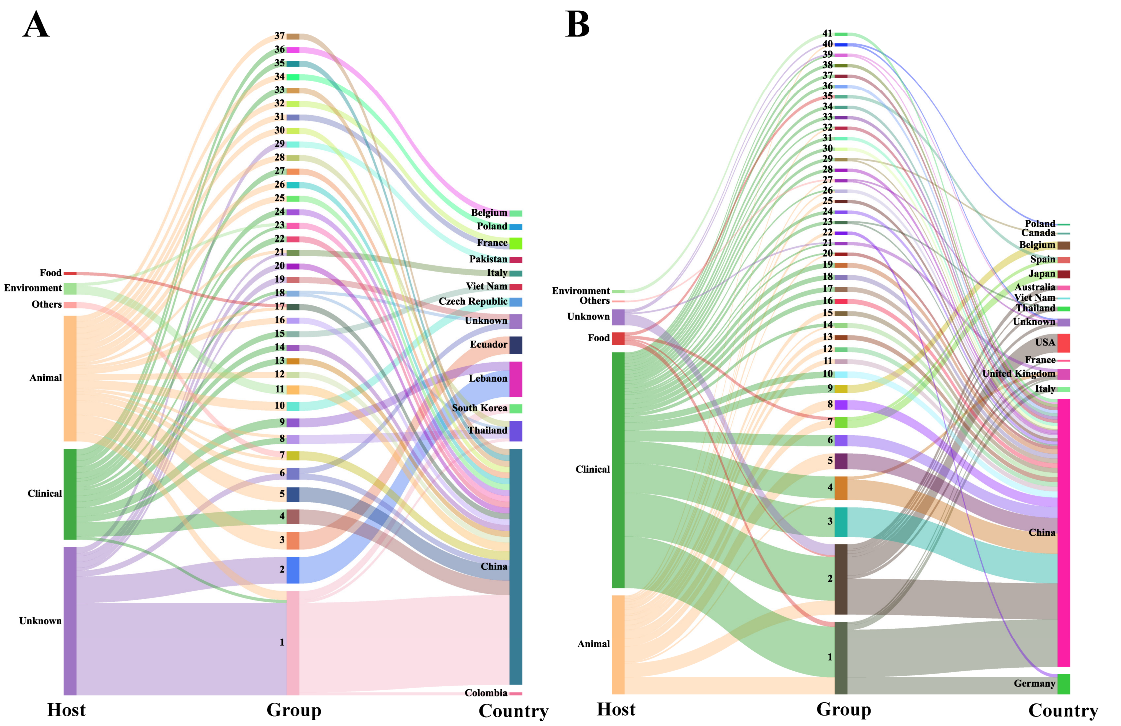

Figure S8. Distribution of each SNP cluster group of ST10 E. coli (A) and ST34 S. enterica (B) across host sources and countries.

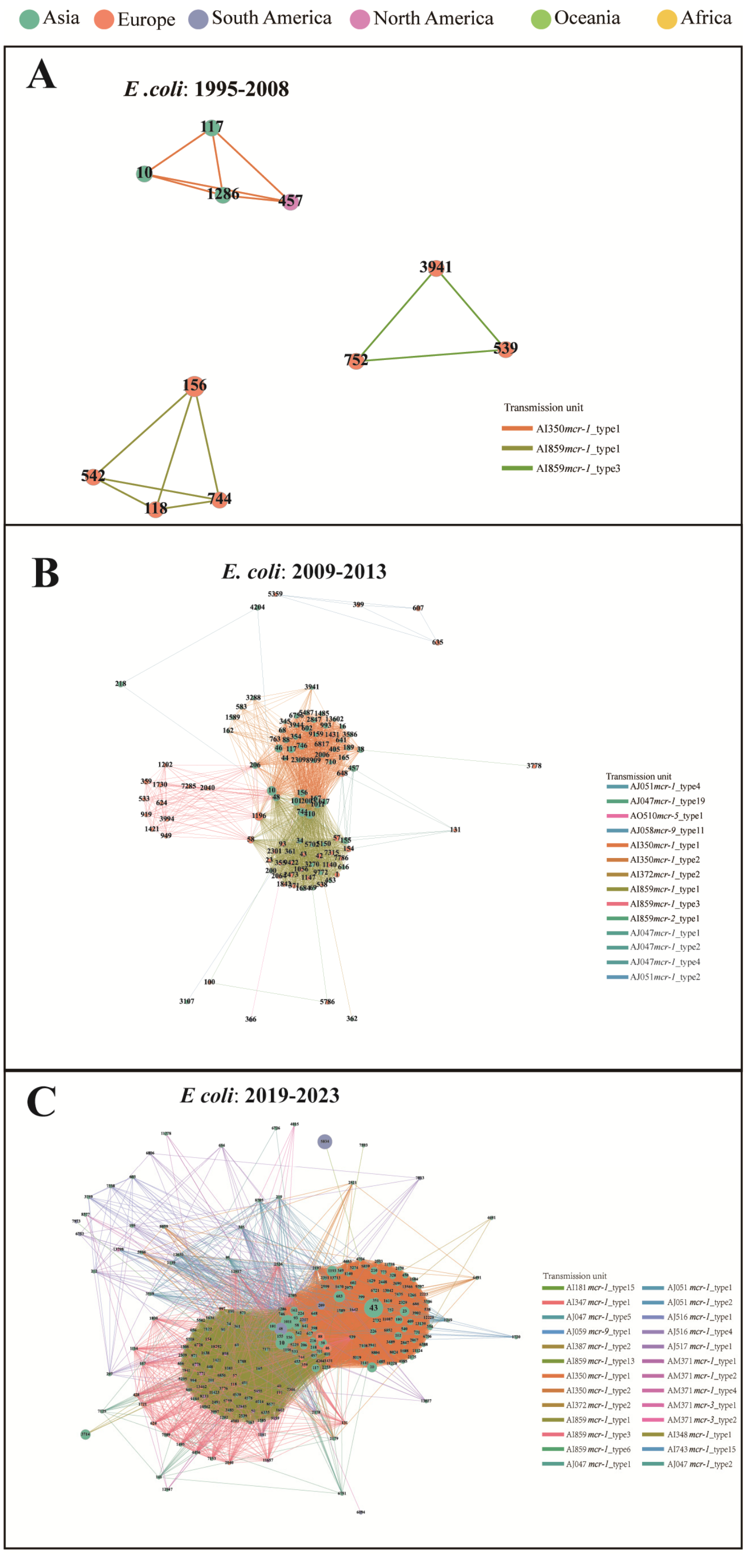

Figure S9. Transmission network of *mcr*-harboring plasmid across *E. coli* STs in the three periods: (A) 1995-2008, (B) 2009-2013, and (C) 2019-2023.

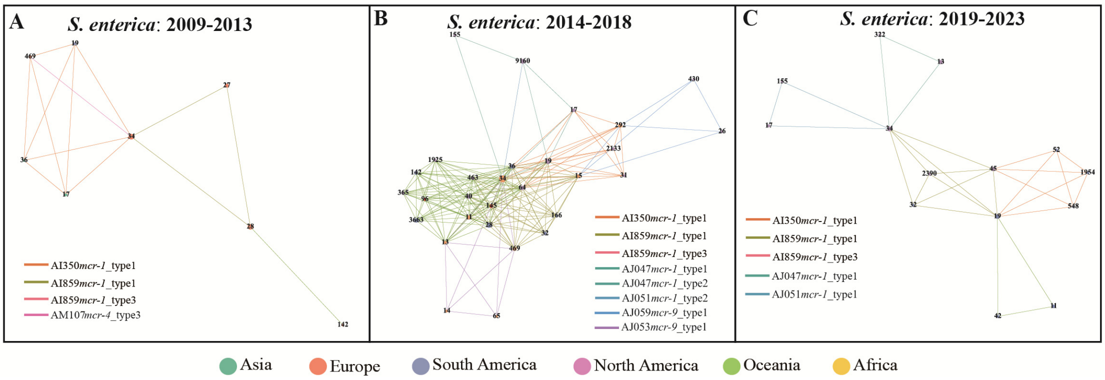

Figure S10. Transmission network of *mcr*-harboring plasmid across *S. enterica* STs from 2009 to 2023.

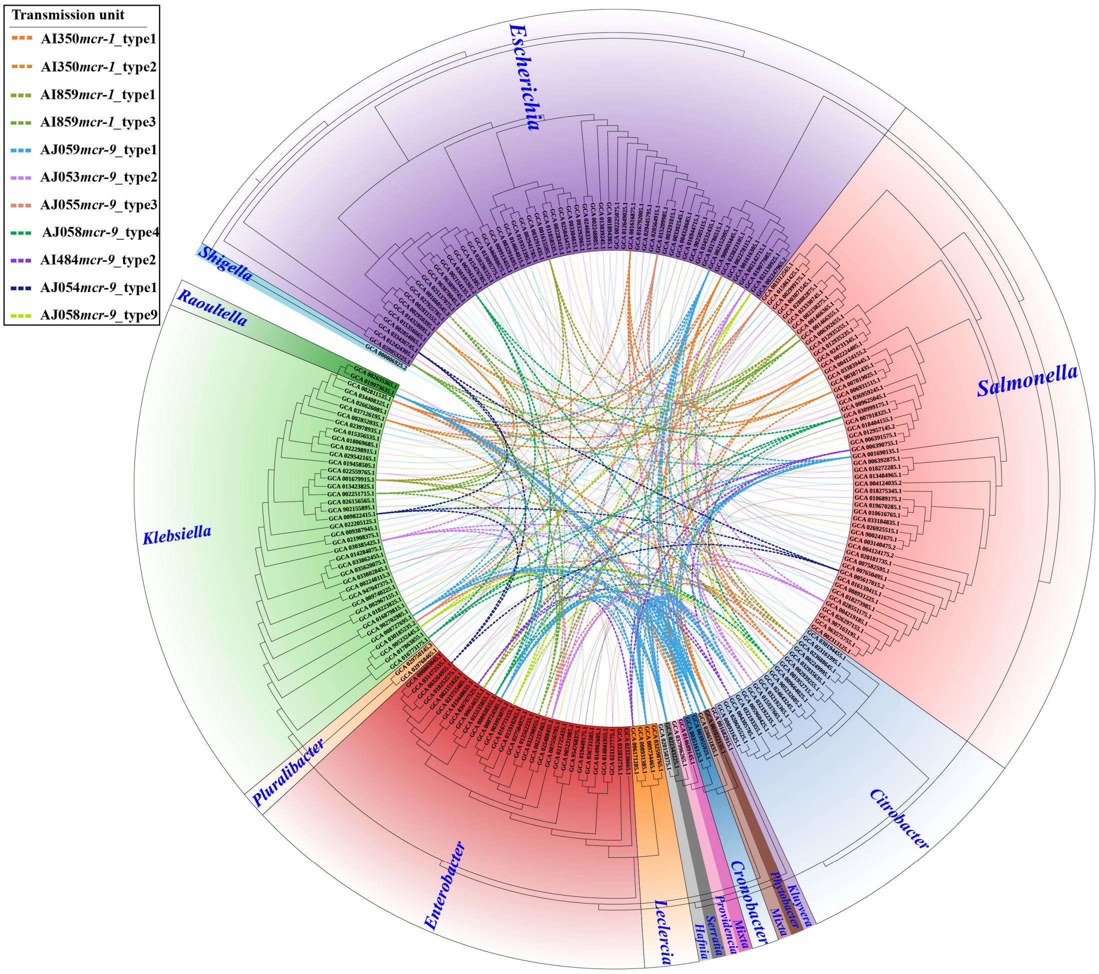

Figure S11. Cross-genus transmission network of *mcr*-harboring plasmids. The phylogenetic tree is constructed based on 120 conserved marker genes extracted by GTDB-TK software. The connecting line between tree tips indicates putative transmission events, with line colors representing different transmission units and line styles indicating transmission range (solid lines: < 4 genera; dashed lines: ≥4 genera). The transmission units spanning ≥ 4 genera are marked at the top-left position.

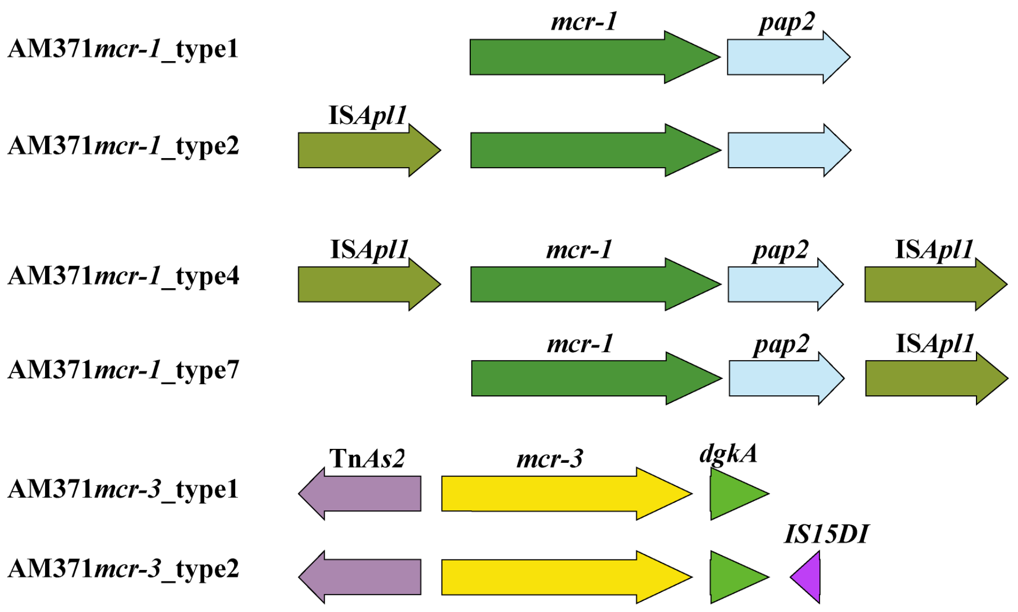

Figure S12. Genetic environment of *mcr*-flanking genes in IncP1 plasmids. The different genes are represented by different colors, and the gene name is marked at the top position. The transcription direction of the gene is represented by the arrow.
